# Effect of Formaldehyde and Curcumin on Histomorphological Indices, Gene Expression Associated with Ovarian Follicular Development, and Total Antioxidant to Oxidant Levels in Wistar Rats

**DOI:** 10.1155/2023/4662440

**Published:** 2023-02-01

**Authors:** Zahra Farshad, Abbas Shahedi, Farzaneh Fesahat, Azam Hassanpour, Morteza Anvari

**Affiliations:** ^1^Department of Biology & Anatomical Sciences, Shahid Sadoughi University of Medical Sciences, Yazd, Iran; ^2^Reproductive Immunology Research Center, Shahid Sadoughi University of Medical Sciences, Yazd, Iran; ^3^Medical Nanotechnology and Tissue Engineering Research Center, Shahid Sadoughi University of Medical Sciences, Yazd, Iran

## Abstract

The present experimental study was undertaken to investigate the effect of formaldehyde (FA) and curcumin (CUR) on histomorphological features, antioxidant potential, and messenger ribonucleic acid (mRNA) levels of genes related to follicular development in FA-exposed rats. 24 Wistar female rats were divided into four study groups and given intraperitoneal injections of FA (10 mg/kg) (*N* = 6), FA (10 mg/kg) + CUR (100 mg/kg) (*N* = 6), sham (*N* = 6), and control (*N* = 6) for 14 days. Ovarian follicular histology, the related gene expression, blood factors, and anti/oxidation potentials were assessed using ovarian tissue and serum, respectively. The *klotho* was significantly overexpressed in the FA group compared with controls and shams. Contradictory, *the factor in germ line alpha* was significantly down-regulated in FA and FA + CUR groups compared to shams and controls. A significant decline was seen in the number of ovarian follicles in the FA group, independent of the developmental stage. Regarding the comparison of the FA + CUR group to other groups, a significant change was seen in the number of secondary, graafian, and atretic follicles. The FA group demonstrated significantly lower hemoglobin, red blood cell count, hematocrit, and mean corpuscular hemoglobin concentration than controls. The activity of glutathione peroxidase increased significantly in the FA group than in the controls. Despite the deleterious effects of FA on histological and molecular aspects of rat ovarian follicles, CUR does not appear to have a protective effect against the hazardous effects of this chemical. However, CUR in some cases has positive effects such as reducing follicular destruction and interstitial edema.

## 1. Introduction

Formaldehyde (FA), a pervasive environmental pollutant, has long been discussed concerning the environmental policy. The human body or any animal can be exposed to organic solvents such as FA in many situations, including at work, during cleaning, and during excessive use. The three main ways that this chemical is absorbed are through the digestive system, skin, and respiratory system. It is detrimental to various organs in addition to being a potent respiratory system inflammatory trigger [1]. Exposure to FA in the air for a long time has been associated with several reproductive symptoms, including irregular menstruation, genital infections, abortions, low birth weight, and female birth abnormalities [2]. In investigations on animals, exposure to FA harmed the ovarian histology and oocyte structure in female rats and resulted in oocyte apoptosis [3]. According to the previous studies, FA exposure caused deoxyribonucleic acid (DNA) damage to the oocyte as well as apoptosis in the uterus and ovary [4].

Curcumin (CUR) is the main polyphenol in turmeric (curry powder) [5]. Recently, herbal medical compounds have been considered to be investigated for possible treatments. It has been demonstrated that CUR is beneficial to the gonads, testis, and ovaries due to its antioxidant, anticancer, antiapoptotic, and anti-inflammatory properties through multiple mechanisms, such as its effects on the expression profile and the associated signaling [6–8]. By preventing the generation of reactive oxygen species (ROS), CUR can lessen the harm FA causes to the parameters of the sperm and testicular structure [9]. CUR is also a powerful antioxidant that protects cells from damage caused by oxidative stress and biochemical changes [10]. CUR can also decrease oxidative stress in patients with polycystic ovary syndrome due to its anti-inflammatory properties [11, 12].

There are a variety of reports on the potential effects of FA on oogenesis [13], structure and function of the ovary [14], and also beneficial impacts of CUR on premature ovarian failure [15], oocyte apoptosis [12], and polycystic ovary syndrome [16]. However, to the best of our knowledge, this is the first study using a different methodology than others to investigate the effect of either FA or CUR and both together on histomorphological indices, expression of genes associated with ovarian follicle development, and antioxidant potential in the animal model.

## 2. Materials and Methods

### 2.1. Experimental Design

All animal experiments in this study were approved by the Animal Ethics Committee of Yazd University of Sciences (IR.SSU. MEDICINE.REC.1400.121). 24 Female Wistar rats, 8 weeks old, weighing 150–200 gr, were obtained from the animal house of Yazd Institute of Reproductive research, Yazd. Iran. All experiments were performed under standard laboratory conditions. They received water and food while undergoing a separate 12-hour light/dark acclimatization period. All rats were randomly and equally assigned into four experimental groups as follows: (1) The FA group including the rats was intraperitoneally injected with 10 mg/kg FA daily for 14 days [17]. (2) The CUR + FA group including the rats was intraperitoneally injected with 100 mg/kg CUR and 10 mg/kg of FA daily for 14 days [4, 18]. (3) The sham group including rats was intraperitoneally injected with dimethyl sulfoxide (DMSO) solvent daily for 14 days, and (4) the control group included rats that received no injection and were kept in normal conditions.

### 2.2. Administration of FA and CUR and Sample Collections

All Wistar rats in FA and FA + CUR groups were intraperitoneally injected by FA (Sigma Lowis ST, Mo) at a dose of 10 mg/kg. 100 mg/kg of CUR (368 MW. a Da, 94% of purity, Sigma. Louise, MO, USA) was intraperitoneally injected into six rats of FA + CUR group along with FA. 10% DMSO, to resolve the CUR for absorption, was used for sham interventions. After 14 days of injections, all rat ovaries were removed during a midline laparotomy following anesthetization with xylazine and ketamine (100 mg/kg and 10 mg/kg, respectively). Due to obtaining the ovaries, each animal's abdomen was opened. The ovaries were weighed and then collected for further investigation. A sterile syringe was used to obtain blood samples from the heart. All serums were then separated from blood cells by centrifugation at 1500 × *g* for 15 minutes.

### 2.3. Relative Gene Expression Assessments

Genes expression assessments associated with folliculogenesis *(klotho (KI), follicle-stimulating hormone receptor (FSHR), growth differentiation factor-9 (GDF-9), factor in germ line alpha (FIGLA), B-cell lymphoma 2 (BCL-2), and BCL-2-like protein 4 (BAX))* were studied by quantitative real-time-polymerase chain reaction (qRT-PCR). Using a total RNA extraction kit (Parstous Co. Iran), total ribonucleic acid (RNA) was extracted from ovarian tissue while adhering to the manufacturer's instructions. Following that, RNA concentration was determined by using spectrophotometry and set to amounts of 10 ng/ml. The complementary deoxyribonucleic acid (cDNA) synthesis reaction was carried out as follows: 10 min at 25°C, 60 min at 47°C, and 5 min of striking at 85°C to stop the reaction. The cDNA was then used for qRT-PCR using SYBR Green RT-PCR Master Mix and the one-step Applied Biosystems real-time thermocycler. As it is shown in Table 1, the *β-ACTIN* gene was considered as the reference gene. Each PCR run was repeated three times. The RT-PCR was performed as follows: first denaturation phase at 95°C for 10 min, followed by 40 cycles at 95°C for 15 s, 56–60°C (based on the optimal melting temperature set for each primer) for 20 s, and 72°C for 30 s. To perform PCR, a 20 *µ*l reaction mix comprising 1 *µ*l of cDNA was used, with 1 *µ*l of each forward and reverse primer, 12.5 *µ*l of master mix, and 5.5 *µ*l of diethyl pyro carbonate (DEPC) treated water. To standardize the results by eliminating variations in mRNA and cDNA quantity and quality, the *β-ACTIN* transcript was used. The level of gene expression between the treatment groups was quantified using the 2^−∆∆Ct^ method [19].

### 2.4. Stereological Study

#### 2.4.1. Ovary Tissue Preparation

**Figure 1:**
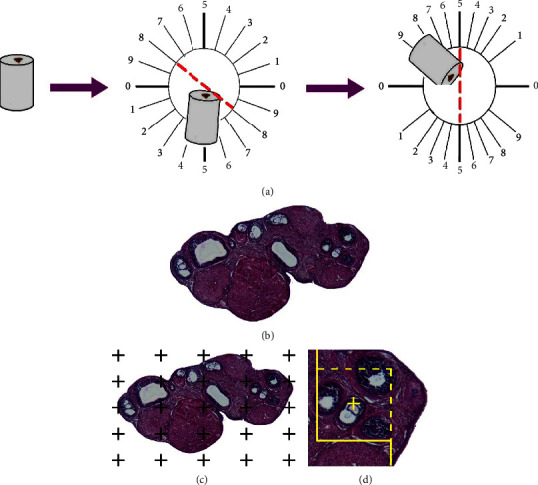
Ovarian cylindrical paraffin block was divided into sections using isotropic uniform random (IUR). (a) Sections of ovarian tissue were stained with hematoxylin and eosin (H&E). (b) The volumes of the cortex, medulla, and corpus luteum were measured using a point-counting technique. (c) The optical dissector method was used to estimate the number of follicles (d).

The prepared ovaries were embedded in cylinder-shaped paraffin blocks and divided into sections using isotropic uniform random (IUR) sections. For this purpose, the paraffin block of the ovary was positioned on the *φ*-clock at random (Figure 1(a)).

The paraffin block was then put on the *θ*-clock, and the division was performed along with the chosen score (Figure 1(a)) [20].

Afterward, 5 and 20 *μ*m sections were cut with a microtome, mounted on slides, and stained with hematoxylin and eosin (H&E).

#### 2.4.2. Determining the Ovarian Size

The volumes of the ovary were assessed by Cavalieri's principle [20]. For this purpose, 8–12 sections per rat were chosen by systematic random sampling. The first section was chosen randomly, and the subsequent sections were gained at the same intervals. The living figures were subjected to the stereology probes by using the point-counting software at the magnification of 30×. The volumes of the ovary were measured using the consequent formula:

(1) $$\begin{array}{c} V\left(ovary\right)=A\times TA=\sum P\left(ovary\right)\times \frac{a}{p}\text{.} \end{array}$$

“∑*P*” was the whole number of points superimposing on the ovarian pictures, *a*/*p* was the area related to each point, and “*T*” is the distance between the chosen sections.

#### 2.4.3. Determining the Volumes of the Cortex, Medulla, and Corpus Luteum

The volumes of the cortex, medulla, and corpus luteum were estimated using sections with 5 *µ*m thickness. The borders of the cortex, medulla, and corpus luteum were identified in each ovary segment (Figure 1(b)). The volume density of the cortex, medulla, and corpus luteum was calculated using the “point-counting approach,” and the following formula after the 386.66× final magnification stereology probes were applied to the live figure (Figure 1(c)) [20, 21]:

(2) $$\begin{array}{c} Vv\left(structure\right)=\frac{\sum P\left(structure\right)}{\sum P\left(total\right)}\text{.} \end{array}$$

The complete points that struck the selected structure in this formula, “∑*P*” (cortex or medulla or corpus luteum)” and “∑*P* (total ovary section),” were the ovary sections, respectively. To calculate the volumes of the cortex, medulla, and corpus luteum, the ovarian thickness was multiplied by “*V* (ovary)”: *V* (structure) = *Vv* (structure) × *V* (ovary).

#### 2.4.4. Estimating the Number of Follicles

The follicular number was determined using the optical dissector technique. An Eclipse microscope (E200, Nikon, Tokyo, Japan) with a high numerical aperture (NA = 1.30) × 40 oil immersion objective was housed in the optical dissector, along with the electronic microcator (MT12, Heidenhain, Traunreut, Germany), for calculating *Z*-axis movement.

The counting frame is a three-dimensional stereological probe that is used in conjunction with a stereology software system to count the number of follicles (Stereolite, SUMS, Shiraz, Iran). At a final magnification of ×386, the unbiased counting frame was placed on the live picture ovary (Figure 1(d)). The guard zone is the area above and below each portion of the ovary. To prevent tissue artifacts, these locations were utilized from cutting through the ovarian sections in this location during tissue processing. The first 3.5 *μ*m of the guard region above or below the follicle was not counted. The “height” of the dissector, in this case, 10 *μ*m, determined the separation between the above and below guard zones.

The follicles were detected, and the nucleus was visible in the greatest focus of the counting frame, was positioned wholly or partially there, and avoided making contact with the banned line of choice (Figure 1(d)) [20, 21]. The following algorithm is used to calculate the follicles' numerical density (NV):

(3) $$\begin{array}{c} Nv\left(\frac{cells}{total}\right)=\frac{\sum Q-}{\sum p\times \left(a/f\times h\right)\times \left(t/BA\right)}\text{,} \end{array}$$

where “Σ*Q*-” represents the number of different follicles located within the height of the dissector, “Σ*P*” represents all counting frames in the microscopic fields, “*h*” represents the height of the dissector, “*a*/*f*” represents the region of the counting frame, “*t*” represents the calculation of the section compactness by the microcator, and “BA” represents the block precession of the microtome. The density of the ovarian section was measured throughout the microscope viewing area using IUR from each section of the ovary. The numerical density (*Nv*) was multiplied by *V* (ovary) to approximate the total number of follicles.

#### 2.4.5. The Classification of the Ovarian Follicles

The following classification was performed to identify each ovarian follicle during the cell count: primordial follicle (the oocyte was enclosed by a single layer of flat granulosa cells). The primary follicle (the oocyte was enclosed by a layer of cubic granulosa cells). The secondary or antral follicle (the oocyte was enveloped by two or three layers of cubic granulosa cells with a space between the cells). The mature or graafian follicle (the oocyte surrounded by more than 4 layers of granulosa cells with C-shaped spaces and cumulus oophorus). The atretic follicle (the oocyte contained apoptotic granulosa cells detachment, oocyte autolysis, and the degeneration of the zona pellucida [20].

### 2.5. Measurement of Total Antioxidant, Oxidant Capacity, and Blood Factors

Blood samples were collected from all groups at the end of the 14 days of administration. Blood was drawn after anesthesia and kept in tubes containing ethylene diamine tetra acetic acid as the anticoagulant for 10 min at room temperature. The sample tubes were then centrifuged at 3500 rpm for 10 minutes. 1.5 ml of isolated blood serum was placed in microtubes. White blood cell count (WBC), red blood cell count (RBC), hemoglobin (HGB), hematocrit (HCT), mean corpuscular volume (MCV), mean corpuscular hemoglobin [22], mean corpuscular hemoglobin concentration (MCHC), platelets (PLT), and lymphocytes (LYM) and neutrophils were quantitatively assessed. The serum levels of glutathione peroxidase-1 (GPx), total antioxidant capacity (TAC), and total oxidant status (TOS) were determined by applying the associated enzyme-linked immunosorbent assay kits (ELISA) (Navand Salamat Co. IRAN) according to the manufacturer's guidelines.

### 2.6. Statistical Analysis

We used GraphPad Prism software version 9 to present graphs. Data analyses were undertaken by using IBM SPSS mean ± standard deviation version 25.00 which was used to represent the obtained data. The data's normality was measured using the Kolmogorov–Smirnov test. Data were compared with the one-way ANOVA (Tukey test). The Mann–Whitney test was utilized for comparison among the two groups, and the Kruskal–Wallis test was utilized for comparison between all groups. To compare the ratios between the groups, the chi-squared test was used. The *p* values <0.05 were regarded as significantly different.

## 3. Results and Discussion

### 3.1. Gene Expression

The expression of *FSHR, GDF-9, BAX, KL, FIGLA*, and *BCL-2* is shown in (Table 2). Transcripts of *FSHR, GDF-9,* and *BAX* showed no change between all groups (*p*=0.64, 0.84, and 0.58, respectively). The mean transcript rate of the *KL* gene was significantly higher in the FA group than in shams and controls (*p*=0.03). However, there was no remarkable difference between the FA + CUR group and other groups regarding the *KL* expression. Compared to controls and shams, a significant down-regulation of the *FIGLA* gene was observed in both FA + CUR and FA groups. FA + CUR and FA groups demonstrated significantly higher levels of *BCL-2* mRNA than the sham/control group. The ratio of *BAX/BCL-2* expression was greater in the FA group, whereas it was balanced in other groups (Table 2).

### 3.2. Histomorphological and Stereological Analysis

**Figure 2:**
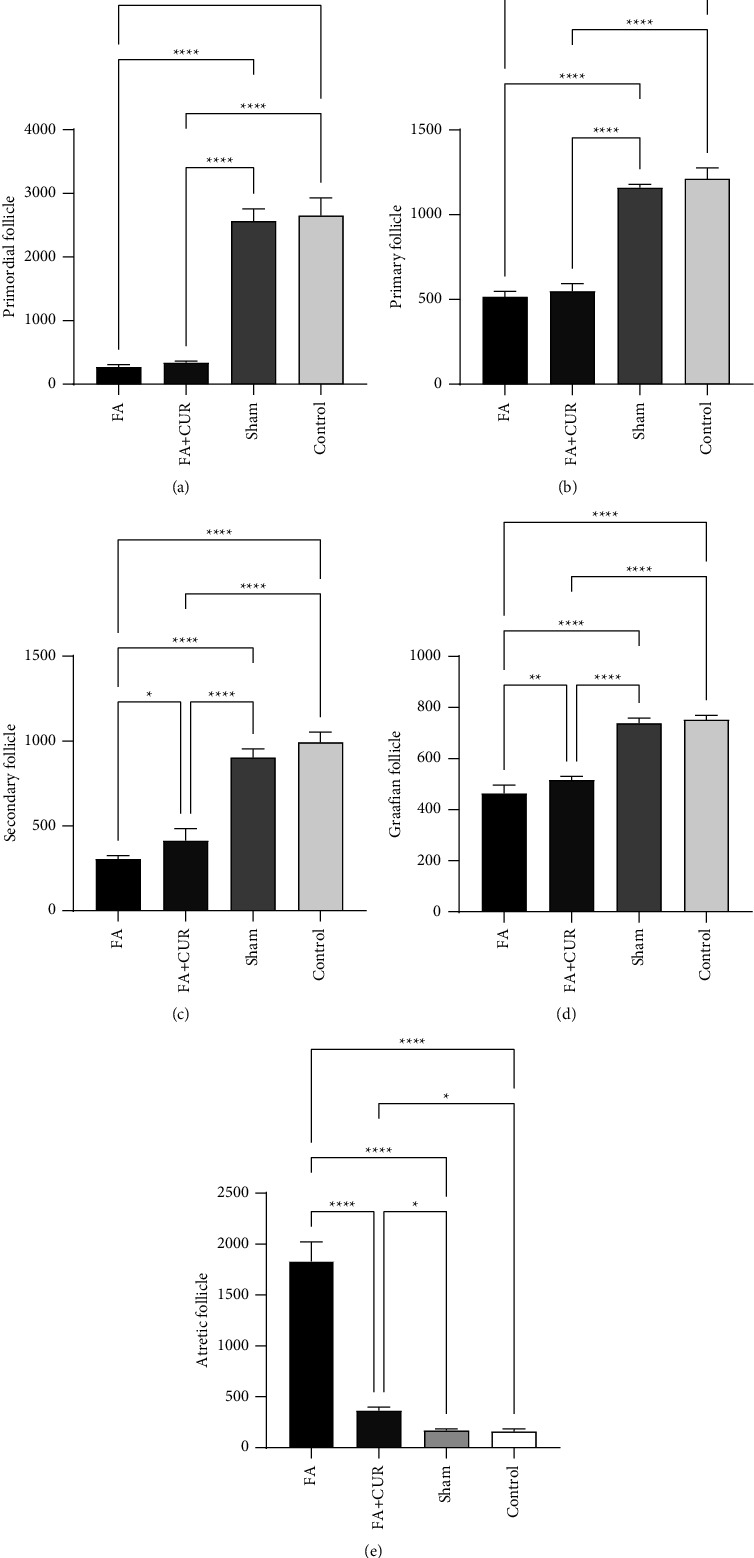
A comparison of the number of ovarian follicles among study groups. Each column presents the mean number ± SD of primordial (a), primary (b), secondary (c), graafian (d), and atretic follicles (e) in study groups. *P* < 0.05 were considered statistically significant. FA: formaldehyde, CUR: curcumin, and SD: standard deviation. ^*∗*^*p*=0.02, ^*∗∗*^*p*=0.009, and ^*∗∗∗*^*p* < 0.0001. We used GraphPad Prism software version 9 to present graphs. Two groups were analyzed using the post hoc (Tukey) test, and all groups were analyzed using the one-way ANOVA test.

Although an 18% decrease was seen in ovarian weight in FA + CUR and FA groups compared to control and sham groups, there were no notable changes in either group. FA + CUR and FA groups did not differ significantly in terms of the volumes of the ovarian cortex, medulla, and corpus luteum (Table 3). In comparison to the controls and shams, the mean number of primordial follicles was significantly reduced in FA + CUR and FA groups (*p* < 0.0001). However, no considerable difference was found between FA + CUR and FA groups (*p*=0.92). A significant decline in the number of primary follicles was observed in FA + CUR and FA groups compared to controls and shams (*p* < 0.0001). No considerable difference was found between FA + CUR and FA groups (*p*=0.61). There was a significant decrease in the mean number of secondary follicles among FA + CUR and FA groups compared to controls and shams (*p* < 0.0001). A significant difference was found in FA + CUR and FA groups (*p*=0.03). The mean number of graafian follicles in FA + CUR and FA groups was significantly lower than in shams and controls (*p* < 0.0001). The results showed that FA + CUR and FA are significantly different from each other in terms of graafian follicles (*p*=0.009). In comparison to other groups, the greatest number of atretic follicles belonged to the FA group (Figure 2).

### 3.3. Ovarian Morphology

**Figure 3:**
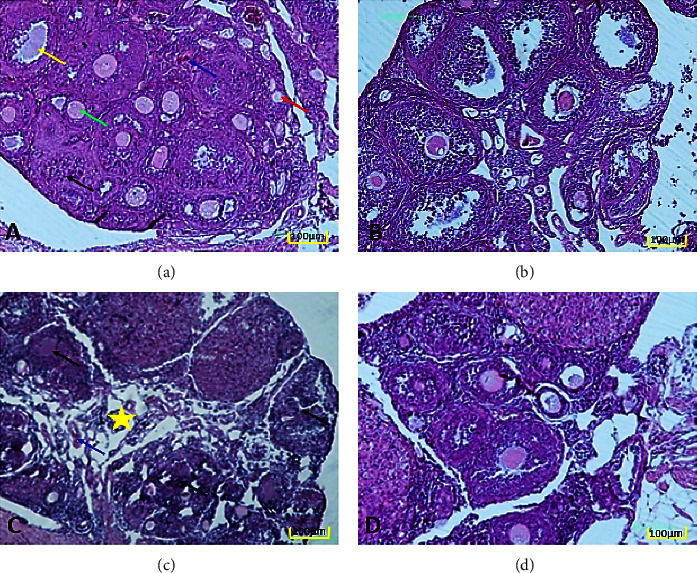
Light micrographs of ovarian tissue (H&E staining). (a) Control, (b) sham, (c) FA, and (d) FA + CUR. Normal histological structure (a, b). Along with a rise in edema in the medulla and vascular hyperemia, more atretic follicles were seen (c). There is less interstitial edema and follicular damage (d). Yellow arrow: graafian follicle, green arrow: secondary follicle, black arrow: atretic follicle, red arrow: primary follicle, and blue arrow: blood vessel; ^*∗*^ovarian medulla with edema. Magnification: 10X/scale bar: 100 *µ*m. H&E: hematoxylin and eosin, FA: formaldehyde, and CUR: curcumin.

A group of follicles at different stages (primordial, primary, secondary, and mature) were seen in the ovary of the control and sham groups (Figures 3(a) and 3(b)). However, in the FA group, an increase in atretic follicles was observed along with an increase in edema in the medulla and vascular hyperemia (Figure 3(c)). In the FA + CUR group, follicular destruction is reduced and interstitial edema is less visible (Figure 3(d)).

### 3.4. Quantification Effects on Blood Parameters

In this study, the FA group showed significantly lower concentrations of RBC, HGB, MCHC, and HCT than controls (*p*=0.03, 0.02, 0.04, and 0.04, respectively). Among the study groups, a significant change in neutrophil counts was seen (*p*=0.03). The most counts of neutrophils were observed between the FA + CUR group and controls (*p*=0.08). There was no noticeable difference in any variable between the other study groups (*p* ≥ 0.05).

### 3.5. Oxidant and Antioxidant Status

Although TAC levels were higher in FA + CUR and FA groups compared to shams and controls, it did not lead to a statistically significant difference (*p* > 0.05). Although there was no remarkable difference between any of the research groups, the GPx level in the FA group was significantly greater than in the controls (*p*=0.005). The TOS level did not remarkably change between the study group (*p* > 0.05). As seen in Table 4, the FA + CUR group represented the highest TAC/TOS ratio.

In this experiment, the effects of FA and CUR on histomorphological features, antioxidant potential, and mRNA levels of genes related to follicular development in FA-exposed rats were investigated. FA environmental exposure occurs mostly by inhalation. However, a lot of earlier animal experiments involved indirect exposure techniques such as intraperitoneal and subcutaneous injections [3, 23]. In the current study, an intraperitoneal injection was to ensure the same intensity and effect of exposure to FA and further treatment with CUR for associated rats and to minimize the error of the study design. One of the treatment techniques to decrease the destructive effects of FA is the use of effective and safe antioxidants.

The findings of this investigation demonstrated that the FA group had higher levels of *KL* expression. This gene is a protective factor against oxidative stress damage, so its up-regulation in the FA group indicates the external induction of oxidative stress. Similarly, several studies have reported that *KL* can attenuate oxidative damage and apoptosis [24, 25].

Our results showed a significant down-regulation of *FIGLA* in FA + CUR and FA groups compared to controls. Following RNA level analysis, it was revealed that *FIGLA* was primarily expressed in mice gonads and that the ovary had a substantially higher abundance of its transcript than the testis. *FIGLA* was primarily expressed in the oocytes of primordial follicles in the ovary, but as the follicle grew, its expression reduced [26]. Despite having normal embryonic gonadal development, female mice lacking *FIGLA* are unable to generate primordial follicles after birth, leading to huge oocyte loss and infertility [27]. Previous research has shown that the lack of *FIGLA* greatly slows down the meiotic process, damages DNA, and causes oocyte death [28]. Taken together, due to the important role of *FIGLA* in folliculogenesis, its low expression in FA + CUR and FA groups could indicate the negative effect of FA on molecular pathways related to folliculogenesis.

Apoptosis is caused by the extracellular factors that control the *BCL-2* and *BAX* genes [29]. Our findings in the number of follicles as well as apoptotic gene expression declared that apoptosis was more pronounced in the groups receiving FA. The findings of FA and CUR on apoptosis-related genes in the ovaries revealed that the ratio of *BAX to BCL-2* was higher in the FA group in comparison with the other groups. However, it seems neither FA nor CUR could not regulate the apoptotic pathway dependent on the *BCL-2* family. These results may be due to the dose and duration that were used in this study. Therefore, it seems that further investigations are needed with different methodologies and other apoptotic-related genes.

Disruption of folliculogenesis, which is shown by a reduction in the number of distinct types of follicles in the ovarian parenchyma and interstitial edema, may consider one of the main adverse consequences of the FA injection. FA is one of several chemical solvents that could damage the ovaries and disrupt follicular growth by impairing corpus luteum function [13].

Rats in the FA group had follicular destruction as well as interstitial edema. This experiment indicates that FA caused most of the follicles to enter the atresia stage. A probable cause of follicular atresia could be an insufficient defense against ROS [30]. Additionally, ROS in the ovary causes alterations in ovarian blood flow and the death of granulosa [31, 32]. On the other hand, the information gathered from the results on the ovary, cortex, and medulla's total volume supported the findings of the investigation of the number of follicles, and we observed a decrease in the volume of the ovary and its subgroups (this decrease in volume was due to follicular destruction), although this decrease in volume was not significant between the groups. These results showed that FA interferes with folliculogenesis, negatively affects the ovarian structure, and leads to oxidative stress. Kareem et al. in 2014 reported similar findings. Based on their results, the number of follicles in the ovaries of rats exposed to FA by inhalation was reduced [13].

Coadministration of CUR with FA compensated for follicular destruction and decreased interstitial edema. According to ovarian structure results, CUR can reduce oxidative stress damage caused by FA to some extent, and this effect was seen in the reduction of atresia follicles in the FA + CUR group. The decline in follicular destruction after injection of CUR at a concentration of 100 mg/kg in rats may be due to its antioxidant and anti-inflammatory properties [33, 34]. Based on the literature, the effects of CUR on FA-induced ovarian damage have not yet been investigated.

Oxidative stress occurs when the oxidative balance is disturbed [33, 34]. The balance of oxidants and antioxidants is essential for the normal biological function of cells and tissues. The ratio of TAC/TOS was higher in the FA + CUR group compared with other groups. Similarly, Wang et al. in 2017 found that CUR can eliminate free radicals and improve antioxidant status in mouse ovaries [35].

## 4. Conclusion

Despite the deleterious effects of FA on histological and molecular aspects of rat ovarian follicles, CUR does not appear to have a protective effect against the hazardous effects of this chemical, at least at the dose and timing used in this study. But in some cases, it has positive effects such as reducing atretic follicles, and increase in secondary and graafian follicles was also observed. According to our data, it could be suggested to work with a different dosage of FA and CUR, a longer duration as well as alternative methods of the intervention such as the gavage.

## Figures and Tables

**Table 1:** Oligonucleotide primers.

Genes	Primer sequence (5′-3′)	Sequence amplified	Product size (bp)
*KL*	F_*GAATACGCAAAGTAGCCACAAAG* R_*GAGCAAGACTCACTGAGGATG*	XM_039089809.1	104
*FSHR*	F_ATCATTTGCTGGATTTGGAGAC R_TTGGGTAGGTTGGAGAACACAT	NM_199237.2	96
*GDF-9*	F_CAATACCGTCCGGCTCTTCAG R_AGGGGTCCTGTCATCTGGTTG	XM_017597502.2	72
*FIGLA*	F_CAGGAAGCCCAGTAAAGTTGA R_CTTCTTTCTTCAAGCCACTTGC	XM_575589.7	218
*BCL-2*	F_CGGGAGAACAGGGTATGA R_CAGGCTGGAAGGAGAAGA	NM_016993.2	149
*BAX*	F_CAGGACGCATCCACCAAGAAG R_TGCCACACGGAAGAAGACCTC	NM_017059.2	135
*B-ACTIN*	F_ CCCATCTATGAGGGTTACGC R_TTTAATGTCACGCACGATTTC	NM_031144.3	150

F: forward, R: reverse, *KL*: *klotho*, *FSHR*: follicle-stimulating hormone receptor, *GDF-9*: growth differentiation factor-9, *FIGLA*: factor in germ line alpha, *BCL-2*: B-cell lymphoma 2, *BAX*: BCL-2-like protein 4, and *B-ACTIN*: beta-actin.

**Table 2:** Comparison of relative gene expression profiles among study groups.

Variables	Formaldehyde^a^	Formaldehyde + curcumin^b^	Sham^c^	Control^d^	*P* value
*FSHR*	1.29 ± 0.45	1.14 ± 0.31	1.28 ± 0.81	1.02 ± 0.15	0.64

*GDF-9*	1.3 ± 0.42	1.03 ± 0.14	0.99 ± 0.5	1.01 ± 0.52	0.84

*KL*	3.05 ± 0.75	2.4 ± 0.85	0.87 ± 0.22	0.81 ± 0.33	0.06 0.6^a,b^ **0.03**^**a,c**^ **0.03**^**a,d**^ 0.19^b,c^ 0.34^b,d^ 0.9^c,d^

*FIGLA*	0.91 ± 0.36	1.44 ± 0.45	3.07 ± 0.34	3.59 ± 0.55	**0.006** 0.44^a,b^ **0.009**^**a,c**^ **0.02**^**a,d**^ **0.02**^**b,c**^ **0.01**^**b,d**^ 0.99^c,d^

*BAX*	1.18 ± 0.35	1.15 ± 0.24	0.74 ± 0.11	1.1 ± 0.33	0.58

*BCL-2*	0.99 ± 0.26	1.01 ± 0.2	2.94 ± 0.74	2.94 ± 0.42	**0.007** 0.91^a,b^ **0.03**^**a,c**^ **0.02**^**a,d**^ **0.01**^**b,c**^ 0.09^**b,d**^ 0.73^c,d^

*BAX/BCL-2*	1.14	1.03	1.11	1.00	—

*FSHR*: follicle-stimulating hormone receptor, *GDF-9*: growth differentiation factor-9, *FIGLA*: factor in germ line alpha, *Kl*: *klotho*, *BCL-2*: B-cell lymphoma 2, and *BAX*: BCL-2-like protein 4. The data were shown as the mean ± SEM. The Mann–Whitney and Kruskal–Wallis tests were carried out, respectively, to compare two and all groups. SEM: standard error of mean. *P* < 0.05 was considered as bold values.

**Table 3:** Comparison of the volume of ovary, cortex, medulla, and corpus luteum among study groups.

Volumes	Formaldehyde^a^	Formaldehyde + curcumin^b^	Sham^c^	Control^d^	*P* value
Ovary	8.67 ± 1.81	9.02 ± 1.006	10.93 ± 3.5	11.76 ± 2.11	0.13
Cortex	8.01 ± 0.53	8.51 ± 0.89	9.69 ± 1.75	9.75 ± 1.05	0.07
Medulla	1.06 ± 0.46	1.3 ± 0.11	1.64 ± 0.53	1.69 ± 0.3	0.06
Corpus luteum	3.02 ± 0.24	3.18 ± 0.2	3.56 ± 0.79	3.79 ± 0.24	0.05

The data were represented by the mean ± SD. Two groups were compared using the post hoc (Tukey) test, and all groups were compared using the one-way ANOVA test. SD: standard deviation.

**Table 4:** Comparison of oxidant and antioxidant status among study groups.

Variables	Formaldehyde^a^	Formaldehyde + curcumin^b^	Sham^c^	Control^d^	*P* value
TOS (*μ*mol/L)	1.49 ± 0.35	1.54 ± 0.37	2.08 ± 0.24	2.18 ± 0.2	>0.05

GPx (mU/ml)	0.005 ± 0.002	0.003 ± 0.0007	0.004 ± 0.0006	0.002 ± 0.0004	**0.006** 0.06^a,b^ 0.62^b,d^ 0.45^b,c^ **0.005**^**a,d**^ 0.59^a,c^ 0.06^c,d^

TAC (mmol Fe^2+^/L)	1.33 ± 0.3	1.08 ± 0.16	0.93 ± 0.04	0.99 ± 0.03	>0.05

TAC/TOS	1.01	**1.93**	1.04	1.001	—

TOS: total oxidant status, GPx: glutathione peroxidase activity, and TAC: total antioxidant capacity. The data were presented as the mean ± SD. The one-way ANOVA test was used to compare all the groups, and the post hoc (Tukey) test was performed to compare two groups. SD: standard deviation. *P *< 0.05 was considered as bold values.

## Data Availability

The data that support the findings of the study are available from the corresponding author on request.
